# Cortisol-induced immune suppression by a blockade of lymphocyte egress in traumatic brain injury

**DOI:** 10.1186/s12974-016-0663-y

**Published:** 2016-08-25

**Authors:** Tingting Dong, Liang Zhi, Brijesh Bhayana, Mei X. Wu

**Affiliations:** Wellman Center for Photomedicine, Massachusetts General Hospital, Department of Dermatology, Harvard Medical School, 50 Blossom Street, Boston, MA 02114 USA

**Keywords:** TBI, T lymphocytes, Cortisol, Inflammation, cAMP

## Abstract

**Background:**

Acute traumatic brain injury (TBI) represents one of major causes of mortality and disability in the USA. Neuroinflammation has been regarded both beneficial and detrimental, probably in a time-dependent fashion.

**Methods:**

To address a role for neuroinflammation in brain injury, C57BL/6 mice were subjected to a closed head mild TBI (mTBI) by a standard controlled cortical impact, along with or without treatment of sphingosine 1-phosphate (S1P) or rolipram, after which the brain tissue of the impact site was evaluated for cell morphology via histology, inflammation by qRT-PCR and T cell staining, and cell death with Caspase-3 and TUNEL staining. Circulating lymphocytes were quantified by flow cytometry, and plasma hydrocortisone was analyzed by LC-MS/MS. To investigate the mechanism whereby cortisol lowered the number of peripheral T cells, T cell egress was tracked in lymph nodes by intravital confocal microscopy after hydrocortisone administration.

**Results:**

We detected a decreased number of circulating lymphocytes, in particular, T cells soon after mTBI, which was inversely correlated with a transient and robust increase of plasma cortisol. The transient lymphocytopenia might be caused by cortisol in part via a blockade of lymphocyte egress as demonstrated by the ability of cortisol to inhibit T cell egress from the secondary lymphoid tissues. Moreover, exogenous hydrocortisone severely suppressed periphery lymphocytes in uninjured mice, whereas administering an egress-promoting agent S1P normalized circulating T cells in mTBI mice and increased T cells in the injured brain. Likewise, rolipram, a cAMP phosphodiesterase inhibitor, was also able to elevate cAMP levels in T cells in the presence of hydrocortisone in vitro and abrogate the action of cortisol in mTBI mice. The investigation demonstrated that the number of circulating T cells in the early phase of TBI was positively correlated with T cell infiltration and inflammatory responses as well as cell death at the cerebral cortex and hippocampus beneath the impact site.

**Conclusions:**

Decreases in intracellular cAMP might be part of the mechanism behind cortisol-mediated blockade of T cell egress. The study argues strongly for a protective role of cortisol-induced immune suppression in the early stage of TBI.

## Background

Acute traumatic brain injury (TBI) is a major cause of mortality and disability in the early decades of life in many developed countries. At least 5.3 million people in the USA currently require long-term or life-long assistance with the activities of daily living after TBI [1]. TBI results in cerebral structural damage and functional deficits due to both primary and secondary injury. The primary injury is caused directly by the external mechanical force at the moment of trauma leading to skull fractures, brain contusions, lacerations, diffused axonal injuries, vascular tearing, intracranial hemorrhages, etc. The primary injury is followed by development of secondary neuronal damage that evolves over a period of months [2], thereby providing a golden opportunity for prevention and intervention. Tremendous efforts have been made in the past decades toward exploring the cellular and molecular mechanisms underlying secondary brain damage as well as identification of specific targets for prevention and/or therapeutics against this disorder [2]. It is now believed that a cascade of molecular, neurochemical, neuronal cell apoptosis, cellular, and immune processes contribute to secondary brain damage as a consequence of mitochondrial dysfunction, cerebral hypoxia, and disruption of calcium homeostasis in cells at the impact site [2, 3].

A growing body of evidence indicates that inflammation induced by primary brain injury plays dual and opposite roles in the outcome of TBI [4]. On one hand, it contributes to reparation and regeneration processes of the primary brain injury, for instance, clearance of necrotic and apoptotic cells by phagocytic cells and promoting neuron growth at the injured site [5, 6]. On the other hand, it facilitates secondary brain injury via the production of various inflammatory cytokines such as interleukin-1-alpha (IL-1α) and interleukin-1-β (IL-1β), tumor necrosis factor alpha (TNF-α), and interleukin-6 (IL-6) [7, 8]. The brain is well known to be an immune privilege site, and infiltration of inflammatory cells to it is largely restricted by the blood-brain barrier (BBB) under a physiological condition [9]. However, TBI often results in an invasion of neutrophils, monocytes, and lymphocytes from the periphery and activation of microglia due to disruption of the BBB. This initiates a cascade of inflammatory responses [10]. Likewise, T lymphocytes have been shown to infiltrate the brain parenchyma post-injury, but their role in the secondary brain injury development following TBI remains poorly understood [11].

Both pre-clinical and clinical studies have shown significant, acute increases of cortisol levels in serum and cerebrospinal fluid in response to TBI [12, 13]. The increased cortisol might suppress inflammation in the brain in order to protect the injured brain tissues from inflammation insult, in light of the well-documented anti-inflammatory function of cortisol, a steroid hormone. The current investigation revealed that an elevated level of serum cortisol was inversely correlated with the number of peripheral lymphocytes, in particular, T cells following brain trauma. Cortisol appeared to sequester lymphocytes in the secondary lymphoid tissues by blocking their egress, contributing to reduced inflammation and cell death at injured brain tissues. The study sheds novel insight into the mechanism underlying cortisol-mediated suppression of inflammation and protective roles of cortisol in TBI at the early stage.

## Methods

### Animals

Eight-week-old female C57BL/6 mice were purchased from Charles River Laboratories and maintained in a 12-h light/dark cycle. All animal experiments were approved by the Institutional Animal Care and Use Committee (IACUC) of the Massachusetts General Hospital and performed according to the National Institutes of Health guidelines for the Care and Use of Laboratory Animals.

### TBI induction

Mice were subjected to a closed head TBI by a standard controlled cortical impact on the left lateral with intact skull and scalp as previously described [7, 14]. In brief, the mice were anesthetized with isoflurane and placed on a mobile plate with their hair removed from the head. A flat face 2-mm diameter tip of the pneumatic impact device (AMS 201, AmScien Instruments, Richmond, VA) was positioned on the left hemisphere center, lowered gradually down to touch the scalp, and recorded as zero depth (sham control). The punch depth was then set 2 mm using a screw-mounted adjustment. A 4.9 ± 0.2 m/s velocity and 80 ms contact time were specified by setting 150 pounds per square inch (psi) for a high pressure and 30 psi for a low pressure impact. These parameters were selected to yield a trauma giving rise to a neurological severity score (NSS) of 3–5 at 1 h post-TBI also called mild TBI (mTBI). After recovery from anesthesia, the mice were returned to cages with post-operative care.

### Quantification of circulating lymphocytes

Blood samples were collected from tail vein in 1 and 4 h after TBI to assess plasma cortisol and circulating lymphocytes or 4 h after hydrocortisone injection (Sigma, 10 mg/kg) to confirm suppressive effects of cortisol on peripheral leukocytes. In separate groups of mice, TBI was induced as above, immediately followed with i.p. injection of either sphingosine 1-phosphate (S1P) (Enzo Life Sciences, 5 μM/kg) or rolipram (Sigma, 30 μM/kg), and blood samples were collected 1 h later. Cells were pelleted, suspended, and treated with ammonium-chloride-potassium (ACK) buffer to lyse erythrocytes. The cells were then counted and stained with PE-anti-CD3 antibody for T cells, APC-anti-CD19 antibody for B cells, FITC-anti-Ly6G antibody for neutrophils, or PE-Cy7-anti-F4/80 antibody for monocytes, followed by flow cytometry analysis on BD FACSAria.

### Quantification of plasma cortisol by liquid chromatography-tandem mass spectrometry (LC-MS/MS)

Quantitative analysis of hydrocortisone in serum samples was performed on an LC-MS/MS instrument. Fludrocortisone acetate was used as a reference standard; known amounts of this compound were added to the serum extract prior to the LC injections. The following working parameters were used for the LC-MS/MS analysis: scan type, MRM (363 → 121 transition for hydrocortisone and 423 → 239 transition for fludrocortisone acetate); polarity, positive; ionization, ESI; column, C18, 2.1 × 50 mm, 1.8 μm; gradient, solution A = acetonitrile, solution B = 10 mM ammonium acetate in water, 20 → 100 % of A over 5 min with a flow rate of 0.4 ml/min.

### Intravital imaging of T cell egress in lymph nodes

T cells were isolated from lymph nodes and spleens of normal C57BL/6 mice and treated with a mixture of rat anti-mouse monoclonal antibodies against CD19, CD32, and CD16 followed by depletion of antibody-bound cells with BioMag goat anti-rat IgG (Polysciences Inc., Warrington, PA) as previously described [15]. The purified T cells were stained with 20 μM 5-(and-6)-(((4-chloromethyl) benzoyl) amino) tetramethylrhodamine (CMTMR, Invitrogen) for 20 min at 37 °C. The labeled cells were adoptively transferred to cognate C57BL/6 mice by tail intravenous injection of 1 × 10^7^ cells per mouse. The recipient mice were then subcutaneously injected with 15 μg anti-LYVE-1 Ab (R&D Systems) conjugated with Alexa Fluor-647 (monoclonal antibody labeling kit, Invitrogen) in a hind footpad, followed by i.p. injection with 10 mg/kg of hydrocortisone or saline 16 h later. After 2 h of hydrocortisone injection, the mouse was anesthetized and placed on an electrically heated plate to maintain the temperature at 36 °C and had their popliteal lymph nodes exposed by a small skin incision. The lymph node to be imaged was bathed with a continuous flow of warm saline in order to maintain a local temperature at 36 °C during imaging. Intravital imaging of the lymph node was performed using a home-built microscope and the images were acquired using an in-house developed software [16]. The in vivo confocal microscope was equipped with three photomultiplier tubes (PMT, Hamamatsu, R9110) which were optimized to provide bright images with a high contrast. Each *x*-*y* plane spanned 250 × 250 μm at a resolution of 2 pixels per μm. Stacks of images were acquired with a *z*-axis resolution of 3 μm per section, and time-series images were obtained in a 20-s interval. To determine whether a cell was inside, outside, or on the border of a cortical sinus, its location relative to the sinusoid wall was assessed in the *x*-*y* and/or the *z* plane. The moving distances and velocities of the tacking cells were tracked for each video segment and calculated using ImageJ software.

### Transwell assay for cell migration

T cell migration was analyzed in 48-well micro chemotaxis chamber (Neuro Probe) as previously described [17]. T cells isolated from normal C57BL/6 mice as above were suspended at 1 × 10^5^ cells in 100 μl in RPMI medium supplemented with 3 % fetal bovine serum (charcoal stripped), 2 mM l-glutamine, 100 U/ml penicillin, 100 μg/ml streptomycin, and 20 μM of either hydrocortisone or vehicle followed by adding the cells to the upper chamber of the transwell. S1P at 20 nM or vehicle was prepared in the same medium and added to the lower chamber of the transwell. Migration was performed for 4 h at 37 °C in a humidified 5 % CO_2_ incubator. The number of migrated cells was determined by counting the cells in the lower chamber.

### S1P administration

S1P (Enzo Life Sciences) was prepared according to the manufacturer’s instructions. Briefly, S1P was dissolved in methanol (0.5 mg/ml) and aliquoted, followed by evaporation of the solvent under a stream of nitrogen to deposit a thin film on the inside of the tube. Prior to use, the aliquots were resuspended in PBS with 4 mg/ml bovine serum albumin (BSA) to a final concentration of S1P at 500 μM. The S1P or the vehicle was i.p. injected into the mice at a dosage of 200 μl per mouse immediately after TBI.

### Measurement of intracellular cAMP

T cells (2 × 10^6^/ml) freshly isolated from normal C57BL/6 mice were incubated at 37 °C in serum free Aim V medium (Invitrogen) and pretreated with 10 μM rolipram (Sigma) or saline for 15 min, followed by a treatment with 100 μM hydrocortisone or vehicle at 37 °C for 5 min. Intracellular cAMP was extracted with hydrochloric acid (HCl) and measured using a cAMP EIA kit following the manufacturer’s instruction (Assay Designs).

### Real-time quantitative reverse transcription polymerase chain reaction (qRT-PCR)

Total RNA was extracted from mouse cortex beneath the impact site 3 days after indicated treatments. The RNA was reverse transcribed with a high capacity RNA-to-cDNA kit (Applied Biosystems, Foster City, CA, USA) and amplified by qRT-PCR) in Roche Lightcycler 480 with a SYBR Green I Master kit (Roche Diagnostics, Indianapolis, IN, USA). The PCR program was preincubation at 95 °C, 5 min, followed by 45 cycles of 95 °C, 10 s, 60 °C, 10 s, and 72 °C, 10 s. The relative levels of each target gene were normalized to endogenous β-actin and calculated using comparative Ct method (ΔΔCt method) [18]. The primer sequences used were 5′-GAAGAGCCCATCCTCTGTGA-3′ (forward) and 5′-TTCATCTCGGAGCCTGTAGTG-3′ (reverse) for IL-1β; 5′-GGCTCAGCCAGATGCAGTTAA-3′ (forward) and 5′-CCTACTCATTGGGATCATCTTGT-3′ (reverse) for CCL2; 5′- GCCGTCATTTTCTGCCTCA-3′ (forward) and 5′-CGTCCTTGCGAGAGGGATC-3′ (reverse) for CXCL10; 5′- GGGCTGGCATTGTTCTCTAATGTC-3′ (forward) and 5′-GGATGGTAGCTGGAAGATCGAAAG-3′ (reverse) for ICAM-1; 5′-GTCTACTGAACTTCGGGGTGAT-3′ (forward) and 5′-ATGATCTGAGTGTGAGGGTCTG-3′ (reverse) for TNF-α; and 5′-CGAGGCCCAGAGCAAGAGAG-3′ (forward) and 5′-CGGTTGGCCTTAGGGTTCAG-3′ (reverse) for β-actin.

### Histological examination

Mice were anesthetized and fixed by cardiac perfusion with cold PBS followed by 10 % formalin. Brains were carefully removed, fixed overnight in 10 % formalin, and subjected to histopathological processing and analysis. Hematoxylin and eosin (H&E)-stained sections of 5-μm-thickness were scanned by Nanozoomer Slide Scanner (Olympus America, Center Valley, PA).

### Immunofluorescence assays

Acetone-fixed tissue sections were incubated with a blocking buffer (3 % BSA, 10 % goat serum and 0.4 % Triton X-100 in PBS) for 1 h at room temperature, followed with primary antibody diluted in the blocking buffer at 4 °C overnight. After reaction with a secondary antibody for 2 h at room temperature and washing, the slides were mounted with DAPI (4′, 6′-diamidino-2 phenylindole)-containing mounting medium (Invitrogen, USA). The primary antibody was rabbit anti-Caspase-3 (active) antibody at a 1:100 dilution (Millipore, USA) and rat anti-CD3 antibody at a 1:100 dilution (BioLegend, USA). TUNEL staining was carried out by an ApopTag® Fluorescein In Situ Apoptosis Detection Kit (Millipore, USA). Images were captured using a confocal microscope (Olympus FV1000, Olympus, Japan). Percentages of Caspase-3+ cells were determined by the number of Caspase-3^+^ cells relatively to DAPI^+^ cells in each field of the 20 randomly selected views of hippocampus area, which represented a total of ten sections from five injured brains in each group. Optical density of TUNEL staining was also calculated in 20 randomly selected views from a total of ten sections from five injured brains in each group by ImageJ software.

### Statistical analysis

The data are presented as mean ± standard errors of measurement (SEM). The statistical analysis was performed using the non-parametric Mann-Whitney *t* test for comparison between two groups and one-way ANOVA or two-way ANOVA for comparison among multiple groups by the Graphpad Prism 6.0 software (GraphPad Software, CA, USA). A value of *P* < 0.05 was considered statistically significant.

## Results

### Elevation of cortisol but reduction of circulating lymphocytes following TBI

**Fig. 1:**
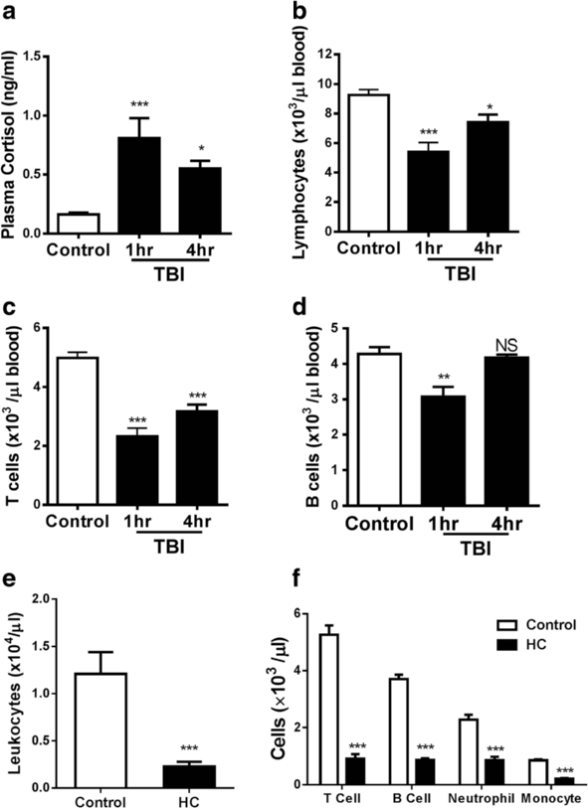
Inverse relationship between cortisol and lymphocytes in blood following TBI. **a** Plasma cortisol was quantified before and 1 and 4 h after TBI. In parallel, the numbers of peripheral lymphocytes (**b**), T cells (**c**), and B cells (**d**) were analyzed at the same time points. A total number of leukocytes (**e**) or indicated cells (**f**) were measured in blood by flow cytometry 4 h after i.p. injection of 10 mg/kg hydrocortisone. Data are expressed as means ± SEM. *n* = 5 in (**a**) or 6 in (**b**, **c**, **d**, **e**, **f**). Significance was determined using one-way ANOVA (**a**, **b**, **c**, **d**) or non-parametric Mann-Whitney *t* test (**e**, **f**). *P < 0.05, **P < 0.01, ***P < 0.001, and NS, no significance compared before and after TBI or HC treatment. The experiment was repeated three times with similar results

Our previous study showed that introduction of inflammation worsened secondary brain damage following mTBI [7]. The mTBI was created by a gentle hit of the brain with an intact skull and scalp by a standard controlled impact, which resulted in extensive cell death at the impact site and significant neurologic severity score (NSS) ranging from 3 to 5 [7]. However, the abnormality was fully recovered functionally and histologically in 4 weeks [7], resembling the majority of mTBI in humans [7]. To determine contributing factors to the full recovery of mTBI, we measured plasma cortisol and found that this steroid hormone rose sharply 1 h post-TBI and declined thereafter (Fig. 1a), similar to what has been reported in patients suffering from traumatic injury or after surgery [12, 13]. In parallel to the elevated level of plasma cortisol was a transient but significantly diminished number of peripheral lymphocytes, with a 42 % decrease in 1 h after injury and a 20 % decrease in 4 h as compared to control mice (Fig. 1b). The decrease appeared to be more predominant in T cells than in B cells, with a 53 % decrease of T cells (Fig. 1c) compared to only a 28 % decrease of B cells (Fig. 1d) at 1 h post-injury. There were no significant differences in the number of circulating monocytes and neutrophils compared to controls at these time points examined. The finding that transient lymphocytopenia is inversely correlated with the amount of plasma cortisol in the animals is consistent with the well-documented immune suppression of cortisol [19].

### Exogenous hydrocortisone depresses the number of leukocytes in the periphery

The inverse correlation between plasma cortisol and the number of circulating lymphocytes following mTBI raised an intriguing possibility that plasma cortisol might be directly responsible for TBI-induced lymphocytopenia. To determine this, mice were intraperitoneally administered hydrocortisone at a dose of 10 mg/kg followed by enumeration of circulating leukocytes. As shown in Fig. 1e, exogenous hydrocortisone reduced the number of leukocytes by 81 % in the periphery over the control mice in 4 h after administration. The reduction was most profound in T cells followed by B cells, neutrophils, and monocytes, all of which are key cellular components in the inflammatory cascade (Fig. 1f). These results corroborate that the reduced number of peripheral lymphocytes is ascribed directly to an elevated level of endogenous cortisol triggered by TBI.

### Hydrocortisone blocks T cell egress from the cortical sinus in lymph nodes

**Fig. 2:**
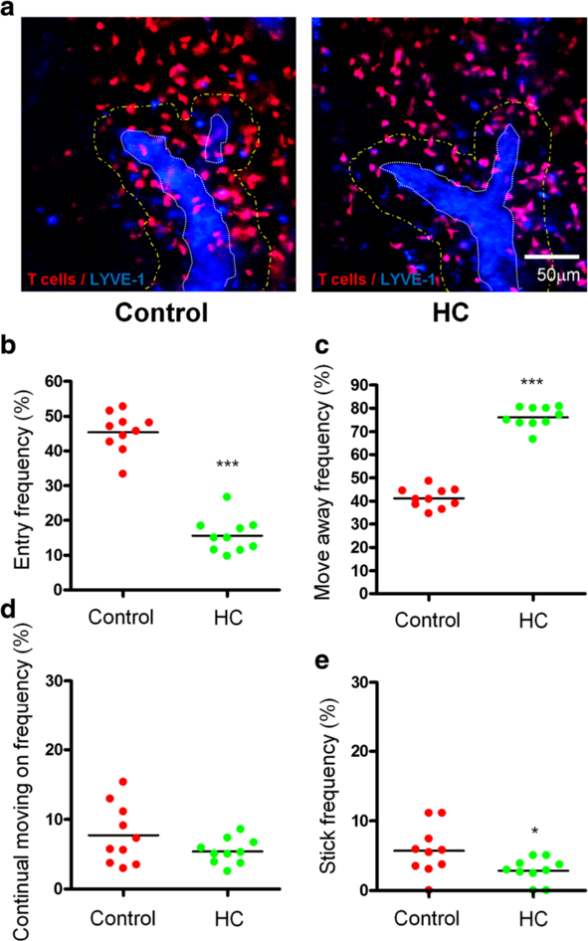
T cell egress is blocked by hydrocortisone. The representative images taken from control or hydrocortisone (HC)-treated mice are shown in (**a**). LYVE-1^+^ cortical sinuses are shown in *blue* pseudocolor in order to distinguish them with CMTMR labeled T cells (*red*) and the representative sinus area is delineated by a *dotted white line*. The *dotted yellow line* outlines the area within 30 μm of distance from the outer boundaries of cortical sinuses. Note: few T cells within cortical sinus in the presence of HC. *Scale bar*, 50 μm. Frequencies at which T cells entered (**b**), moved away (**c**), crawled on (**d**), or stuck to (**e**) (kept adhering to one point on the sinus wall and never displaced during the imaging period after they engaged the sinus) the cortical sinuses in control and HC-treated mice were calculated by manually tracking individual cells in each time-lapse image, with a total of 200 cells randomly selected in 10~15 imaging stacks. Each *dot* represents data from a single time-lapse image, and *bars* represent the means. Significance was measured using non-parametric Mann-Whitney *t* test. *P < 0.05, ***P < 0.001 in the presence or absence of hydrocortisone. Data are combined from two independent experiments each with two lymph nodes imaged in each treatment. The experiment was repeated two times with similar results

**Fig. 3:**
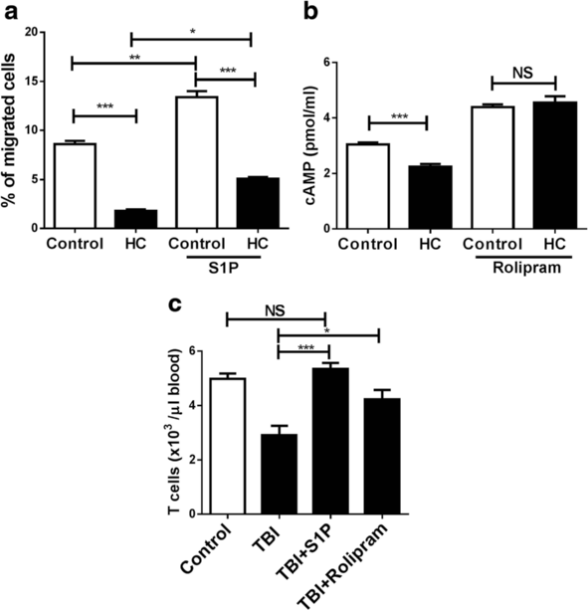
S1P or rolipram increases peripheral T cells in TBI mice. **a** T cell migration was analyzed in 48-well micro chemotaxis chamber, with 20 μM hydrocortisone or vehicle in the upper chamber and 20 nM S1P or vehicle in the lower chamber. The number of migrated cells was assessed 4 h later in the lower chambers. **b** T cells were pretreated with 10 μM rolipram or saline for 15 min and then with 100 μM hydrocortisone or vehicle treatment for 5 min, after which intracellular cAMP level was measured. **c** Peripheral T cells were measured before and 1 h after TBI. S1P or rolipram was i.p. injected immediately after TBI. Results are expressed as means ± SEM. *n* = 9 for (**a**), 6 for (**c**), or 4 for (**b**). Significance was determined using two- (**a**, **b**) or one-way (**c**) ANOVA. *P < 0.05, **P < 0.01, ***P < 0.001, and NS, no significance compared between indicated groups. The experiment was repeated three times with similar results

Although cortisol is well known as a suppressant of inflammation, the underlying mechanism is not fully understood. Previous studies with ^51^Cr-labeled lymphocytes suggested that a decrease in egress of lymphocytes, rather than increased homing or cell death, was the mechanism for the lymphopenia induced by traumatic stress [20]. In support of this, flow cytometric analysis of peripheral T and B cells after propidium iodide (PI) staining did not reveal any significant difference in cell death in the mice (data not shown). We questioned whether cortisol blocked lymphocyte egress, lowering the number of peripheral lymphocytes as did immune suppression drug FTY720, an analog of S1P [15, 21, 22]. We thus tracked T cell egress in part because T cells were key contributors to the acute phase of brain injury [23] and the cells appeared to be more affected by cortisol. To this end, purified naive T cells were labeled with a red vital fluorescent dye CMTMR and infused into cognate mice followed by subcutaneous injection of LYVE-1 antibody to mark lymphatic vessels. The cortical sinusoid region in and adjacent to T cell zones of the popliteal lymph node was imaged 2 h later after hydrocortisone injection by intravital confocal microscopy as we previously described [15]. As can be seen in Fig. 3a, the number of T cells was severely reduced within the cortical sinusoid in the presence compared to the absence of hydrocortisone (Fig. 2a). Consistent with this, when tracking 200 cells in 10~15 randomly selected imaging stacks, we found that the frequency of T cells entering cortical sinusoids diminished to 15 from 45 % in the presence compared to the absence of hydrocortisone (Fig. 2b). On the contrary, T cells moving away from the sinusoids increased from 40 to 75 % in the mice (Fig. 2c). It can be envisioned that as a majority of T cells are moving away from the sinusoids, their egress could be largely prevented, explaining only few T cells within the sinusoids (Fig. 2a) and a reduced number of T cells in the periphery (Fig. 1f). Cortisol also reduced the ability of T cells to adhere on the sinusoids (Fig. 2e), in a good agreement with a low entry frequency (Fig. 2b), because T cell sticking to the sinusoid facilitated entry of the cell into a sinusoid [15]. During T cell egress, T cells continuously move toward and crawl along the sinusoid to search for a “hot entry port” and upon finding the “port,” the cell enters the sinusoid via it [24, 25], but many of them move away from the sinusoid prior to reaching it or after several attempts to associate with or adhere on the sinusoids [15, 26]. Hydrocortisone appeared not to affect the number of T cells that crawled on the sinusoids (Fig. 2d) but greatly increased the number of T cells moving away the sinusoids (Fig. 2c).

### S1P or rolipram increases the number of peripheral T cells after TBI

We went on to determine whether a high level of S1P, an egress-promoting agent, could override cortisol-mediated blockade of T cell egress. We first assessed T cell migration toward S1P in the presence or absence of hydrocortisone in vitro, an assay that is commonly used for assessing S1P function [27]. T cells, along with hydrocortisone or vehicle, were added to the upper chamber and S1P or vehicle was included in the lower chamber of the transwell. As can be seen in Fig. 3a, S1P significantly increased migration of T cells into the lower chamber in the presence or absence of hydrocortisone, suggesting that a high level of S1P may overcome the inhibitory effect of hydrocortisone and restore the number of circulating T cells in mice with mTBI. Indeed, when mice were i.p. administered S1P immediately after TBI, the number of T cells was completely normalized in the blood 1 h post-S1P injection in TBI mice (Fig. 3c). In light of a well-established role for S1P in egress of lymphocytes, the result corroborates the ability of cortisol to block T cell egress, leading to a diminished number of lymphocytes in circulation immediately after mTBI. Moreover, the result also confirmed the ability of hydrocortisone to vigorously blunt T cell migration in the presence or absence of S1P (Fig. 3a), implicating that cortisol hampered T cell egress via an intrinsic signaling pathway of T cells, probably via regulation of cAMP degradation, a key secondary messenger molecule signaling downstream of the S1P_1_ receptor as depicted in Fig. 6. Our previous investigation showed that FTY720 blocked T cell egress by persistent activation of heterotrimeric Gαi proteins leading to prolonged inhibition of cAMP production, apart from induction of S1P_1_ receptor internalization [15]. We therefore measured cAMP after hydrocortisone treatment and found that hydrocortisone lowered cAMP levels significantly (Fig. 3b). The low level of cAMP induced by hydrocortisone was reversed by rolipram (Fig. 3b), a cAMP phosphodiesterase inhibitor that prevents cAMP degradation, corroborating an antagonistic effect of rolipram on cortisol-mediated reduction of cAMP, probably via the same target or the same signaling pathway as illustrated in Fig. 6. In support, i.p. injection of rolipram immediately after mTBI also significantly increased the number of T cells in the periphery, albeit to a much lesser degree in comparison with S1P (Fig. 3c). The results clearly suggest that hydrocortisone blocks T cell egress via a downstream target of the S1P_1_ receptor.

### A protective role for cortisol in TBI pathogenesis

**Fig. 4:**
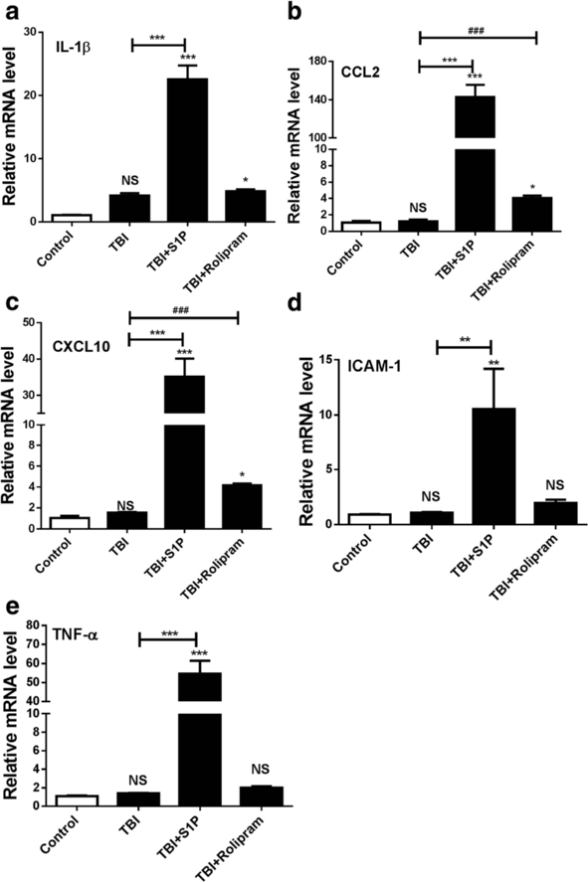
S1P or rolipram exaggerates inflammatory responses in injured brain. IL-1β (**a**), CCL2 (**b**), CXCL10 (**c**), ICAM-1 (**d**), and TNF-α (**e**) were analyzed at the impact site of the cerebral cortex in 3 days after TBI by qRT-PCR. The data are expressed as means ± SEM and normalized to β-actin. *n* = 5, significance was measured using one-way ANOVA. *P < 0.05, **P < 0.01, ***P < 0.001 and NS, no significance compared between indicated groups. ^###^ *P* < 0.001 compared between TBI and TBI + rolipram in CCL2 and CXCL10 expression level by non-parametric Mann-Whitney *t* test. The experiment was repeated three times with similar results

**Fig. 5:**
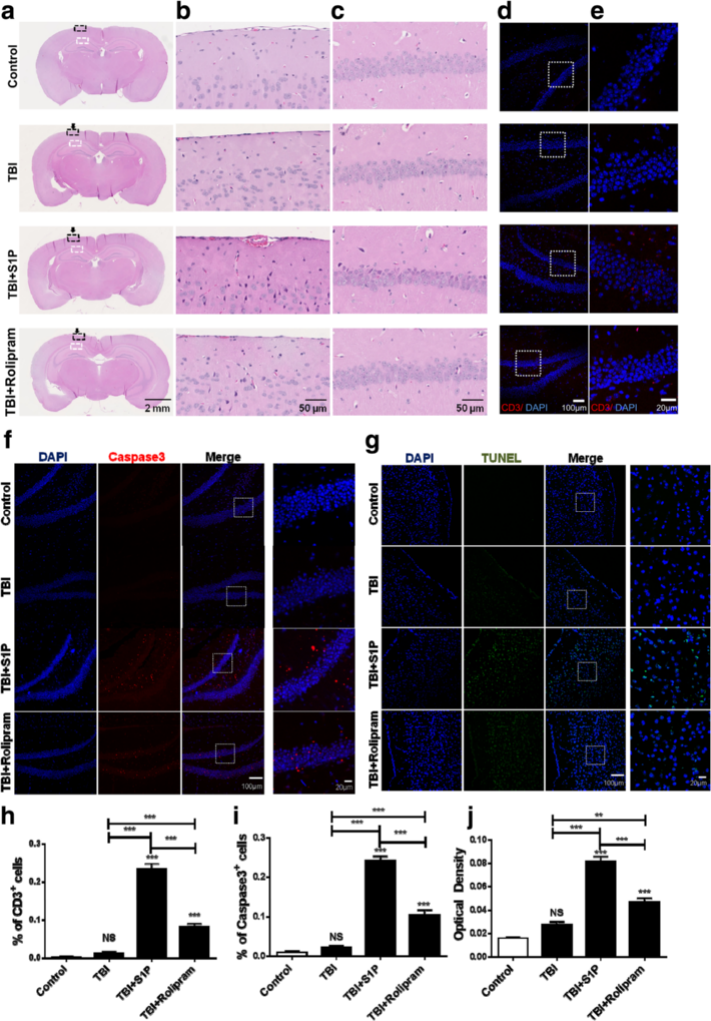
A protective role for cortisol in TBI pathogenesis. **a** Histologic examination of normal control and injured brain at 7 days after TBI with or without administration of S1P or rolipram. The impact site was pointed by an *arrow*. The region of the cerebral cortex was highlighted in a *dashed black line square* and enlarged in panel (**b**); and the hippocampus was outlined by a *dashed white line square* and magnified in panel (**c**). Representative results of six mice in each group. **d** Representative immunofluorescence results of anti-CD3 antibody staining at hippocampus beneath the injured site and enlarged in panel (**e**). **f** Representative immunofluorescence staining for Caspase-3 expression at hippocampus beneath the injured site. **g** Representative TUNEL staining for apoptosis cells at the injury site. Percentages of CD3-positive cells in panel (**e**), Caspase-3-positive cells in panel (**f**), and optical density of TUNEL staining in panel (**g**) were determined by ImageJ and expressed as means ± SEM in (**h**), (**i**), or (**j**), respectively. *n* = 6, significance was measured using one-way ANOVA. *P < 0.05, **P < 0.01, ***P < 0.001 and NS, no significance compared between indicated groups. The experiment was repeated three times with similar results

We next verified positive correlations of circulating lymphocytes with inflammation occurring at the impact site of the brain and directly associated the low inflammation at the injured site with cortisol-mediated blockade of lymphocyte egress in mTBI mice. To this end, several inflammatory mediators, including IL-1β, CCL2, CXCL10, ICAM-1, and TNF-α, were assayed by qRT-PCR at the impact site 3 days after mTBI in the presence or absence of SIP or rolipram [7]. Our previous study showed that mTBI up regulated proinflammatory mediators at 6 h and dwindled down gradually [7]. Consistent with this, transcription levels of these proinflammatory mediators in the mice were not significantly different from controls (Fig. 4). In contrast, S1P robustly bolstered all five inflammatory mediators at the impact sites, confirming a positive relationship between the number of circulating lymphocytes and inflammatory responses occurring at the impact brain tissues (Fig. 4 vs Fig. 3c). Moreover, out of the five inflammatory mediators tested, CCL2 and CXCL10 were also produced at levels significantly higher in TBI mice given rolipram than those mice given vehicle control or uninjured mice (Fig. 4a–c). Histologically, we observed no overt alterations in the gross morphology or at a low magnification on day 7 after injury either in presence or in absence of S1P or rolipram (Fig. 5a). But a robust increase in the number of morphologically abnormal cells was evidenced in the cerebral cortex (B) and hippocampus (C) beneath the injured site in TBI mice receiving S1P compared to TBI controls under a high magnification (Fig. 5b, c). Notably, healthy cell nuclei were relatively large consisting of several discernible nucleoli in the nucleoplasm in the cerebral neocortex and hippocampus in the absence of S1P or normal control mice (Fig. 5b, c). In contrast, morphologically abnormal cells were characterized by dark red staining of the nucleoplasm with eosin and presented only at the injured site (Fig. 5b, c, the third pannel). Although the types of these abnormal cells were unknown, probably both neurons and glias, the cells appeared undergoing apoptosis as revealed by two apoptotic markers, Caspase-3 and TUNEL staining. S1P significantly elevated Caspase-3 activation in the hippocampus (Fig. 5f, i) and TUNEL staining in the cerebral cortex (Fig 5g, j) in comparison with TBI only or controls. When rolipram was given, morphologically abnormal cells were also increased, but largely limited to the cerebral neocortex (Fig. 5b, c bottom). The apoptosis cells were also found both in the cortex (Fig 5g, j) and hippocampus (Fig. 5f, i) in TBI mice receiving rolipram albeit to a much lesser extent in comparison with S1P, consistent with less effect of rolipram on T cell egress in vivo (Fig. 3c). The increase of cell death at the injured site of the brain was proportionally correlated with T cell infiltration in the tissue as revealed by anti-CD3 antibody staining (Fig. 5d, e, h). T cells were hardly presented in the uninjured control mice or mice with mTBI, in agreement with a complete recovery of the injury in mTBI mice. However, the number of T cells increased robustly in the injured brain after i.p. injection of S1P and to a much lesser degree rolipram, as a consequence of elevating levels of T cells in circulation. The results conclude that a high level of peripheral lymphocytes can directly contribute to the heightened inflammation at the injured site of the brain in the early phase of TBI.

## Discussion

Brain has been viewed as an immune-privileged organ with little immunological and inflammatory activity under a physiological condition. This is primarily attributed to the relative impermeability of the blood-brain barrier (BBB) to cellular and molecular components of the immune and inflammatory reactions. However, upon brain injury, both immediate and secondary dysfunctions of the BBB occur as a consequence of disrupting the tight junction complexes and the integrity of the capillary basement membranes [9]. Neutrophils can be found aggregated in the microvasculature as early as 2 h post trauma [28]. Their infiltration in damaged neural tissue commences within 24 h [29], followed by macrophages within 36–48 h after trauma [30]. T lymphocytes have been shown to infiltrate the brain within 2–3 days post injury in a rat TBI model [31]. In those studies, severe or moderate TBI was induced via opening scalp and skull and infiltration of inflammatory cells was apparent [28–31], which is likely to be detrimental and associated with a severe loss of brain tissue and permanent impairment of cognitive neuron function [32]. Cortisol-mediated suppression of inflammation alone may be too weak to be effective in severe TBI. In contrast, mTBI was generated in our study with an intact scalp and skull and overt infiltration of inflammatory cells was not observed, which might be ascribed primarily to cortisol-mediated blockade of lymphocyte egress. In support of limiting inflammation at the injured site by cortisol-mediated blockade on lymphocyte egress at the initial phase of TBI, when the blockade was abolished by administering S1P, the number of circulating T cells was elevated significantly and positively correlated with increasing inflammatory responses (Fig. 4), T cell infiltration, and cell death (Fig. 5) at the impact site. The cortisol-mediated immune suppression observed in this TBI model is highly relevant to what happens in humans as a majority of mTBI recovers fully in humans in a few weeks. The observation hints that immediate immune suppression following TBI can prevent secondary brain damage and thus is beneficial to mTBI patients.

Pre-clinical and clinical studies have supported the use of methylprednisolone, a glucocorticoid drug, as an acute neuroprotectant after acute spinal cord injury [33, 34]. Supplement with hydrocortisone post trauma also improves neurological recovery and leads to beneficial outcomes [35]. Moreover, progesterone, an indirect precursor of cortisol, has shown promise to be a neuroprotective agent, and it is currently under clinical trials for the treatment of TBI [36, 37]. The benefit of inhibiting T cell egress by cortisol is also consistent with a better outcome of cerebral ischemia in T cell-deficient mice than in wild-type controls [38]. Moreover, lymphocyte-deficient Rag1^−/−^ mice are profoundly protected from stab wound injury of the cortex [39]. Apparently, the linkage between lymphocyte infiltration and adverse outcome post-TBI contradicts the key role of T cells in the reparative process. Several studies have shown that T cells are required for neurogenesis and depletion of T cells impairs neuronal cell proliferation [5, 40]. Perhaps, dynamic regulation of the timing and degree of lymphocyte infiltration is pivotal for its neuroprotection. Yet, despite the beneficial role, excess cortisol has adverse effects on mood, cognition, and neurodegeneration [41, 42]. It is thus necessary to monitor cortisol levels post injury and give it preferably to patients with corticosteroid insufficiency [41]. Alternatively, suboptimal FTY720 or anti-S1P antibody may be used to suppress lymphocyte egress at the early phase of TBI to prevent secondary brain damage [43, 44].

**Fig. 6:**
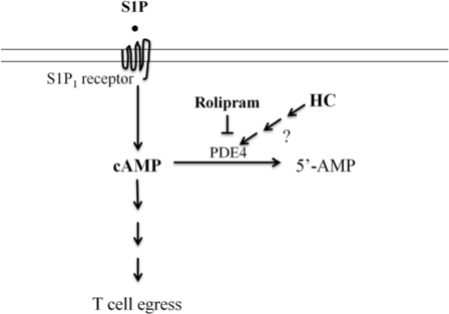
Schematic illustration of a possible mechanism underlying cortisol-mediated blockade of T cell egress. cAMP is one of the important second messengers downstream the S1P_1_ receptor and its production takes central part in the control of T cell egress. One of the cortisol (HC) activities may activate cAMP phosphodiesterase (PDE4) either directly or indirectly and enhance degradation of cAMP to 5′-AMP. Cortisol-facilitated degradation of cAMP may be one of the mechanisms where cortisol compromises T cell egress in the presence of S1P. On the contrary, rolipram inhibits PDE4, leading to increased levels of cAMP and promoting T cell egress

Cortisol is widely recognized for its role in the stress response and for its physiologic anti-inflammatory effects. The mechanism underlying its anti-inflammatory effects may be multifaceted including transcriptional suppression of proinflammatory genes [45, 46] and inhibition of the functions of macrophages and neutrophils [47], and the like. Exogenous glucocorticosteroid administration, especially in supraphysiological doses, also induces cell death of immature T and B cells, but mature T cells and activated B cells are resistant to cell death induced by cortisol at this low dose [48]. Because the number of circulating T cells could be restored in traumatic mice by S1P or rolipram (Fig. 3c), cortisol-induced T lymphocytopenia following TBI was unlikely ascribed to cell death. Our study demonstrating a blockade of T cell egress by cortisol adds a novel mechanism to our current understanding of the anti-inflammatory activity of this steroid hormone. Substantial evidence has shown that T cell egress is initiated by binding of S1P to the S1P_1_ receptor [21, 49]. The S1P_1_ receptor is a G protein-coupled receptor and activates exclusively heterotrimeric Gαi proteins that inhibit adenylate cyclase, leading to brief reduction of cAMP production followed by normalization and increases of cAMP in the cells (Fig. 6) [50]. On the contrary, FTY720 binds to the S1P_1_ receptor and causes the receptor internalization and prolonged reduction of cAMP, which promotes a sinus-moving away signal and blunts T cell egress [15]. The level of cAMP was lower in the presence than in the absence of hydrocortisone (Fig. 3b) but it was elevated by rolipram. Because rolipram can partially overcome the inhibitory effect of cortisol and increase cAMP levels in the presence of cortisol (Fig. 3b), cortisol may activate cAMP phosphodiesterase (PDE4) either directly or indirectly (Fig. 6). The secondary messenger cAMP is a signaling target downstream the S1P_1_ receptor, and thus, hydrocortisone inhibits T cell migration (Fig. 3c) or egress (Fig. 3c), at least in part, by lowering cAMP level in the cells independent of the S1P_1_ receptor.

## Conclusions

We report here that following mTBI, plasma cortisol levels are significantly and transiently elevated, which appears to be directly responsible for the brief lymphocytopenia in the periphery by its ability to block lymphocyte egress from secondary lymphoid tissues. Abrogation of cortisol action on lymphocyte egress by injection of S1P or rolipram was associated with prolonged and increased inflammatory responses and elevated cell death and T cell infiltration at the injured site of the brain cortex, concluding that lymphocyte infiltration of brain in the early phase of brain injury is detrimental. The current work highlights a protective role of cortisol-induced immune suppression in the early phase of TBI and offers valuable information with respect to prevention of TBI soon after injury by a blockade of lymphocyte egress.

## Abbreviations

TBI: Traumatic brain injury
mTBI: Mild traumatic brain injury
S1P: Sphingosine 1-phosphate
LC-MS/MS: Liquid chromatography-tandem mass spectrometry
cAMP: Cyclic adenosine monophosphate
IL-1α: Interleukin-1-alpha
IL6: Interleukin-6
TNF-α: Tumor necrosis factor-alpha
BBB: Blood-brain barrier
NSS: Neurological severity score
ACK: Ammonium-chloride-potassium
HCl: Hydrochloric acid
BSA: Bovine serum albumin
H&E: Hematoxylin and eosin
DAPI: 4′, 6′-diamidino-2 phenylindole
PBS: Phosphate-buffered saline
qRT-PCR: Quantitative reverse-transcription polymerase chain reaction
TUNEL: Terminal deoxynucleotidyl transferase dUTP nick end labeling
CMTMR: 5-(and-6)-(((4-chloromethyl) benzoyl) amino) tetramethylrhodamine
PMT: Photomultiplier tubes
SEM: Standard errors of measurement
HC: Hydrocortisone
PI: Propidium iodide
IL-1β: Interleukin-1-beta
CCL2: Chemokine (C-C motif) ligand 2
CXCL10: C-X-C motif chemokine 10
ICAM-1: Intercellular adhesion molecule 1
PDE4: Phosphodiesterase
